# Accumulation of Anthocyanins and Other Phytochemicals in American Elderberry Cultivars during Fruit Ripening and its Impact on Color Expression

**DOI:** 10.3390/plants9121721

**Published:** 2020-12-07

**Authors:** Yucheng Zhou, Yu Gary Gao, M. Monica Giusti

**Affiliations:** 1 Department of Food Science and Technology, The Ohio State University, 2015 Fyffe Rd, Columbus, OH 43210, USA; zhou.1140@buckeyemail.osu.edu; 2 Department of Extension, The Ohio State University, 2120 Fyffe Rd, Columbus, OH 43210, USA; 3 OSU South Centers, The Ohio State University, 1864 Shyville Rd, Piketon, OH 45661, USA

**Keywords:** polyphenols, fruit development, natural food colorant, *Sambucus canadensis*

## Abstract

American elderberry (*Sambucus canadensis*) is a plant native to North America with anthocyanin-rich fruits. Our objective was to investigate the effects of cultivar and ripeness on the phytochemical characteristics of its fruits and the corresponding color performance. Cultivars ‘Adams’, ‘Johns’, ‘Nova’, ‘Wyldewood’, and ‘York’ were examined for their °Brix, pH, anthocyanin (pH-differential method), and phenolic content (Folin-Ciocalteau method). Extract composition were analyzed by uHPLC-PDA-MS/MS. Color and spectra were determined using a plate reader. All characteristics evaluated were significantly affected by ripeness and cultivar, except for °Brix and total phenolic content, which did not vary significantly among cultivars. Most anthocyanins (63–72%) were acylated with *p*-coumaric acid, with cyanidin-3-(trans)-coumaroylsambubioside-5-glucoside the most predominant. The proportion of acylated anthocyanins was the only characteristic evaluated that decreased during ripening (from 80 to 70%). Extract from fully-ripened fruits exhibited red (*l*_vis-max_ ~520 nm) and blue hues (*l*_vis-max_ ~600 nm) at acidic and alkaline pH, respectively. Extracts from half-ripe fruit rendered yellowish tones and overall dull color. C-18 semi-purified extracts displayed higher color saturation (smaller L* and larger C*_ab_) than crude extracts. The vibrant and broad color expression of fully-ripened fruit extract, especially after C-18 purification, suggests this North American native plant as a promising natural colorant source.

## 1. Introduction

Elderberry (*Sambucus* spp. L.) is a large perennial shrub or small deciduous tree in the Adoxaceae family with purplish-black small fruits that mature in late August to September in different parts of the USA [1]. It is distributed mostly in the temperate and subtropical regions of the Northern Hemisphere and includes 9 to 40 species depending on the taxonomy [1,2,3]. The most economically important species is European elderberry (syn. *S. nigra* L. subsp. *nigra*), which has been widely cultivated in the world from Europe to North America and East Asia [2]. Its fruits have been used as natural food colorants, and used to produce jam, jellies, yogurt, and wine [4]. In the USA, American elderberry (syn. *S. nigra* L. subsp. *canadensis* [L.] Bolli) is the most important domesticated species [3]. The first American elderberry cultivar ‘Adams’ was released in the 1920s, with few additional cultivars developed since then [4], mostly from the New York and Nova Scotia Agricultural Experiment Station breeding programs [1], and the Missouri State University and the University of Missouri Elderberry Improving Program [5].

American elderberry cultivars have shown to have higher yield in the midwestern states in the USA than their European counterparts [6]. A life cycle assessment of these American elderberry cultivars could reveal lower labor and pesticide input due to greater adaptability by following a similar assessment method on wine production [7]. This is because American elderberry can be completely pruned to the ground level in March for a more concentrated harvest in late summer to early autumn, thereby leading to a higher harvest efficiency and less carbon dioxide emission from tractors used for spraying and harvest. Complete removal of old shoots in American elderberry also help control elder shoot borer [8].

Elderberry has been traditionally used as a medicinal crop in many indigenous cultures [4]. The therapeutic effects of elderberry extracts, including flu symptoms alleviation, blood pressure regulation, diabetes and obesity control, and immune system enhancement, have been confirmed in a number of in vitro and in vivo studies [9,10]. Researchers attribute its medicinal and nutritional benefits to its abundant polyphenol content, composed mostly of anthocyanins, flavonols, phenolic acids, and proanthocyanidins [9]. Those polyphenols are known to be high in antioxidant capacity and possess antiviral, antibacterial, and antifungal activities [9]. The demand for valuable natural sources of antioxidant compounds has been going for decades, and this market is expected to gain an annual growth of ~6% globally during the period of 2019–2029 [11], forecasting a considerable growth potential of attention for elderberry in the coming years.

Anthocyanins contribute to both the high antioxidant capacity and the dark-violet color of elderberry fruits. These water-soluble natural pigments have been widely applied as food colorants since they are viewed as safer than synthetic colorants [12]. According to the FDA CFR, fruit (21CFR73.250) and vegetable (21CFR73.260) juice concentrates are approved for use as natural food colorants, but their practical application is restricted by their stability and color shades. Acylated anthocyanins is expected to have enhanced stability to pH, heat treatment, and light exposure than non-acylated anthocyanins through intramolecular copigmentation and self-association [13]. Vegetable sources, such as red cabbage and radish, are usually more abundant in acylated anthocyanins, but they may impart undesirable aromas or flavors to food products [13]. Anthocyanin profiles of European and American elderberry were previously compared, and acylated anthocyanins were only found in American elderberry in considerable quantity [1].

European elderberries have been evaluated for their anthocyanin and phenolic composition [1,14]; and the stability of their anthocyanin extract to heat [15,16], light [15,16], and processing [17]; as well as coloring strength [18]. Due to its high pigment content, European elderberry concentrate has been commercialized as a food coloring agent in the E.U. Despite its unique anthocyanin profile, American elderberry has not been as extensively studied as its European counterpart [18]. Our knowledge regarding American elderberry anthocyanins and phenolics is primarily related to their composition, with little research on their practical applications. Considering its high acylated anthocyanin content and milder flavor, American elderberry can potentially be a promising source of natural color. Its phytochemicals, mostly anthocyanins, may vary among cultivars and during ripening, and this variation could ultimately affect the colorimetric and spectrophotometric properties, therefore merit a more systematic examination.

Our objectives were to investigate the impact of cultivar and ripeness on the accumulation of anthocyanins and other phytochemicals in American elderberry, as well as the color performance of their anthocyanin extracts under a wide pH range. Our research aimed to reveal valuable information to help shape the potential application of American elderberry extract as a natural colorant for food industry.

## 2. Results

### 2.1. Phytochemical Attributes of Different Cultivars

Significant differences in pH, monomeric anthocyanin content, polymeric color, and anthocyanin/phenolic ratio were observed among five cultivars (*p* < 0.05) (Table 1). American elderberry had a pH between 4.5 and 4.9, with ‘Wyldewood’ having significantly lower pH than that of others. The monomeric anthocyanin content varied between 354 and 581 mg C3GE/100 g FW, and the anthocyanin/phenolic ratio was between 0.61 and 0.84, with ‘Johns’ and ‘York’ obtaining the highest and the lowest on both attributes, respectively. However, significantly higher percentage of polymeric color was detected in ‘York’ (10.8%) than in ‘Johns’ (5%), partially explained by their different monomeric anthocyanin content. The °Brix ranged from 12.0 (in ‘Nova’ and ‘Wylderwood’) to 13.1 (in ‘York’) and the total phenolic content was between 582 (in ‘York’) and 707 mg GAE/100 g FW (in ‘Johns’) with no significant differences among them.

### 2.2. Color Development and Phytochemicals Accumulation during Ripening

Elderberry fruits reached a visibly darker surface color when fully ripe, as denoted by the significantly smaller L* (lightness) value of the fully-ripened fruits (L* = 19.1 ± 0.5) compared to the half-ripe counterpart (L* = 23.5 ± 0.5). The mean a* and b* values of the fully-ripe fruits were determined to be 2.4 ± 0.2 and 0.3 ± 0.2, respectively, similar to those of the half-ripe fruits (a* = 2.4 ± 0.2; b* = −0.2 ± 0.1).

**Figure 1:**
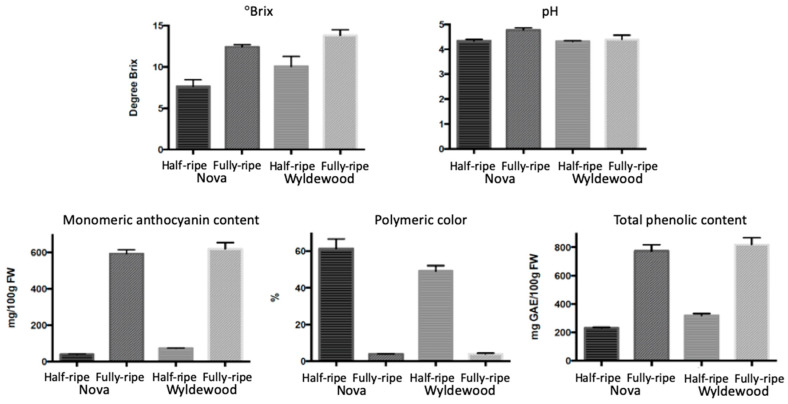
Comparison of ‘Nova’ and ‘Wyldewood’ fruits’ phytochemical attributes (°Brix, pH, monomeric anthocyanins, polymeric color, total phenolic content) at the half and fully-ripe stages. Results expressed as mean ± SD (*n* = 3).

°Brix, pH, anthocyanin, and phenolic content all significantly increased during fruit ripening except the pH of ‘Wyldewood’ (Figure 1). °Brix increased ~4% in both ‘Wyldewood’ and ‘Nova’ during ripening. Half-ripe berries were low in monomeric anthocyanin content, with means of 39 and 72 mg C3GE/100 g FW of ‘Nova’ and ‘Wyldewood’, respectively, consisting with the less intense redness of the fruits, but later greatly increased to 593 and 619 C3GE/100 g FW when fully ripe. Although the total phenolic content multiplied almost threefold during ripening, its rate of increase was not as high as that of the anthocyanin content. For this reason, the contribution of anthocyanins to total phenolic content was modest at the half-ripe stage, but accounted for almost 80% when ripe. Polymeric color proportion was the only monitored attribute that decreased during ripening.

### 2.3. Major Anthocyanins in American Elderberry

**Figure 2:**
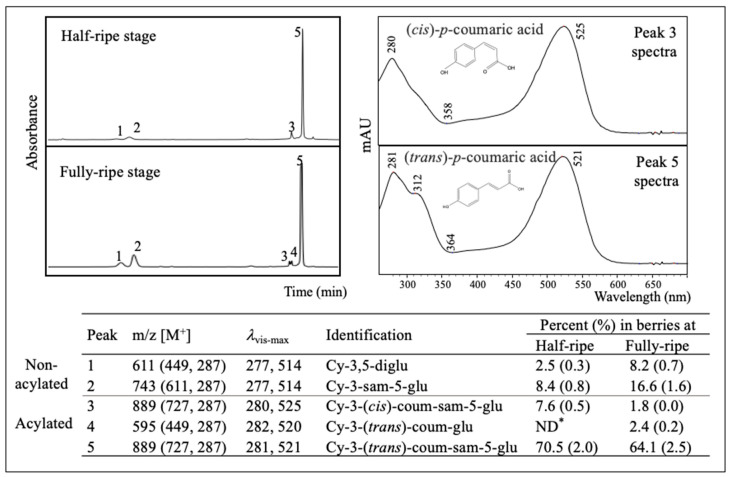
HPLC chromatograms of half and full-ripe ‘Nova’ extracts detected at 520 nm, their peak identifications, and quantifications, and the UV-Vis spectra of peaks 3 and 5. Cy: Cyanidin; glu: glucoside; sam: sambubioside; coum: coumaroyl. *ND: Not detected.

Major anthocyanins in the extract from fully-ripe fruits (FRFE) (listed in order of elution, Figure 2) were identified as cyanidin-3,5-diglucoside (peak 1), cyanidin-3-sambubioside-5-glucoside (peak 2), cyanidin-3-(*cis*)-coumaroylsambubioside-5-glucoside (peak 3), cyanidin-3-(*trans*)-coumaroyl-glucoside (peak 4), cyanidin-3-(*trans*)-coumaroylsambubioside-5-glucoside (peak 5) according to their visible spectra, MS data, and retention times. The extract from half-ripe fruits (HRFE) presented the same anthocyanins with the exception of peak 4. All anthocyanins identified were cyanidin-derivatives, with cyanidin-3-(*trans*)-coumaroylsambubioside-5-glucoside being the most predominant, representing 65–70% of the total anthocyanin content. The *cis* isomers of cyanidin-3-coumaroylsambubioside-5-glucoside (peak 3) were present at both ripening stages, accounted for 7.6% (in HRFE) and 1.8% (in FRFE) of the peak area. The *cis* isomers eluted earlier than its *trans* counterpart showed lower absorption at 310–360 nm, and displayed a larger *l*_vis-max_ (~525 nm). *p*-Coumaric acid was the only acylation found in American elderberry anthocyanins. Acylated anthocyanins constituted ~80% of the total pigments at the half-ripe stage, and ~70% when fully ripened. Nevertheless, ripened berries were much more abundant in both acylated and non-acylated anthocyanins due to their higher total anthocyanin content.

### 2.4. Anthocyanin Profile of Different Cultivars

Different cultivars had similar anthocyanin profiles with minor differences in the proportion of individual peak areas. Among all cultivars tested, ‘Wyldewood’ was overall higher in non-acylated pigments but contained the lowest proportion of the *trans*-isomers (*p* < 0.05) (Table 2). ‘Adam’ and ‘York’ had the highest levels cyanidin-3-(*cis*)-coumaroylsambubioside-5-glucoside, while ‘York’ contained a significantly lower percentage of cyanidin-3,5-diglucoside (*p* < 0.05). Nevertheless, a higher percentage of a certain compound in one cultivar does not necessarily represent higher amount since cultivars also varied on total anthocyanin content.

### 2.5. Spectral Properties of American Elderberry Extract under Various pH

**Figure 3:**
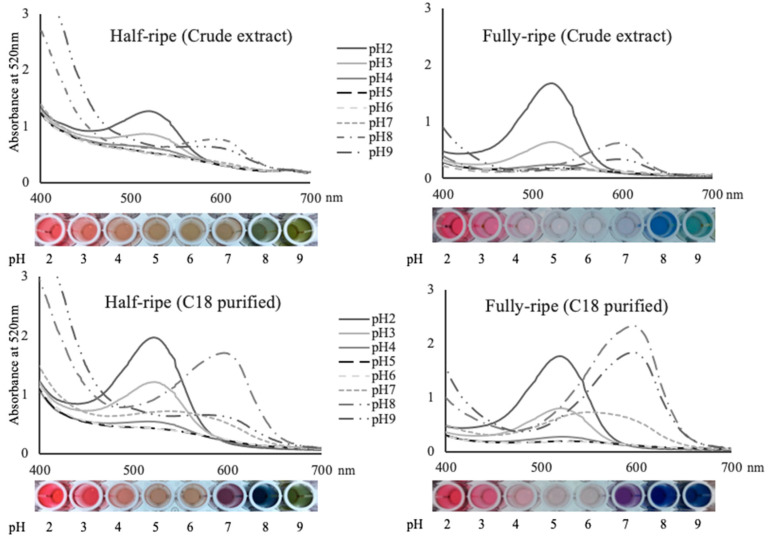
Spectral characteristics of American elderberry ‘Nova’ extracts at two ripening stages before and after C-18 semi-purification in pH 2–9 buffers. Data was collected 1 h after mixing.

All extracts showed similar *l*_vis-max_ ~520 nm at pH 2, but some differences on *l*_vis-max_ were observed at higher pH. Crude HRFE did not show a clear *l*_vis-max_ at pH 4–6, while the *l*_vis-max_ of its fully-ripe counterpart increased from 523 to 549 nm when the pH increased from 4 to 7, although its visible absorbance was weak in this pH range (Figure 3, Table 3). When the pH increased to alkaline region, the *l*_vis-max_ of both HRFE and FRFE shifted to 580–600 nm region. Interestingly, FRFE always obtained larger *l*_vis-max_ than HRFE. The HRFE generally showed higher absorbance at 400–490 nm, rendering orange-yellowish undertones at all pH.

Both HRFE and FRFE exhibited sharper peaks and higher absorbance at their *l*_vis-max_ after C-18 semi-purification, particularly at low and alkaline pH, concurrent with a more vibrant color expression (Figure 3). The shape of the absorption spectra and the *l*_vis-max_ of the HRFE more closely resembled those of FRFE after semi-purification. In spite of this, semi-purified HRFE still showed higher absorbance at 400–490 nm, and lower absorbance at *l*_vis-max_ than FRFE at most pH. Semi-purification of FRFE had a negligible impact on *l*_vis-max_. However, it reduced the absorption between 400–440 nm and increased the absorbance at *l*_vis-max_ notably, particularly under alkaline pH, showing bolder color expression.

### 2.6. Colorimetric Properties of American Elderberry Extract under Various pH

The FRFE expressed colors from red to colorless to purple and blue when the pH increased from 2 to 9 (Figure 3). Under pH 2–3, the FRFE displayed intense red hues with h*_ab_ between 0.4° and 7.3° and C*_ab_ between 28.6–52.6 (Table 3). As the pH increased to mildly acidic (4–6), the L* increased by over 20 units and C*_ab_ were generally small (≤10) due to the deprotonation of flavylium cations, with extracts appearing almost colorless. When the pH further increased into the alkaline region, a purple-bluish color appeared due to the formation of quinonoidal bases, exhibiting h*_ab_ between 249.2–341.8°, and C*_ab_ increased back to 17.5 at pH 8.

The h*_ab_ of HRFE was between 31.9° (red)–103.1° (yellow) under the tested pH range, as predicted from our spectral data (Figure 3, Table 3). The HRFE generally had smaller L* and larger C*_ab_ than its fully-ripe counterpart, especially under mildly acidic pH.

The color properties (L*, C*_ab_, h*_ab_) of FRFE before and after semi-purification were similar at acidic pH, while they largely differed at alkaline pH (Table 3). At pH 7–9, the semi-purification resulted in a decrease in the L* of 24.7–34.4 units and an increase in C*_ab_ of 16–31.7 units. Semi-purification also resulted in the shifts of h*_ab_ from 341.8° to 316.2° (purple hues) at pH 7 and from 249.2° to 270.4° (blue hues) at pH 8. The C-18 semi-purification greatly enhanced color intensity and saturation of the extract, rendering bluer color hues under neutral to alkaline pH.

At acidic pH, extracts from all cultivars except ‘York’ showed high similarities on their color properties (ΔE < 5 in a pairwise comparison) (Table 4). At pH 3, the ΔE between the extracts from ‘York’ and other cultivars fell between 7.28 and 8.25, mainly resulting from their different L* (lightness) value as the L* of York extracts (76.13) were significantly lower than others (82.56–83.93, data not shown). Larger ΔE (up to 14.4) was observed when comparing the color of these extracts under alkaline pH. At pH 8, most pairwise ΔE were larger than 5. Different from the findings at acidic pH where the variance mostly came from L* value, the relatively large ΔE at alkaline pH was mainly contributed by Δb* (blue-yellow color) value. At pH 8, the a* values were similar among cultivars, ranging between −5.98 (‘York’) and −7.35 (‘Johns’), while the b* value varied between −7.69 (‘York’) and −21.52 (‘Adam’), leading to an overall large ΔE among cultivars. 

## 3. Discussion

American elderberry fully-ripe fruits had a °Brix of 12.0–13.1 and a pH of 4.5–4.9, similar to the previously reported data [1,19]. The °Brix of American elderberry is comparable to those of European elderberry (8.9–14.6) [1,20] and blueberry (10.3–13.9) [21], but higher than those of blackberry (4.9–8.0) [22] and raspberry (9.4–11.5) [23]. The pH of most berries ranges between 2.8–4.2 [20,21,22,23]. High °Brix and low acidity are usually associated with a mild, pleasant taste. Nevertheless, American elderberry is seldom consumed as fresh fruit and used most extensively as processed food and beverages [2]. This would be ascribed to its rather small fruit size [24], and tart, tangy or bitter sensory attributes brought by its abundant polyphenols contained [25]. Most anthocyanins have negligible color expression at pH ~4.5 as they transited into the colorless hemiketal form, especially for 3,5-glycosides derivatives [26]. American elderberry was abundant in 3,5-diglycosides (~90%) with a fruit pH ~4.5 (Table 1, Figure 2). Its intense dark purplish-black coloration could be explained by the lower pH of the vacuoles, where anthocyanins are usually localized together with considerable organic acids [27]. The pH of grape vacuole has been reported to be ~1 unit lower than the pH of grape pulp [27]. Similarly, the pH of American elderberry vacuoles is expected to be lower than the fruit pH, therefore protects the integrity of anthocyanins.

Relatively large variability in anthocyanin and phenolic content within the cultivar was observed in both this and previous studies, where berries were sorted by their surface color ahead of phytochemical analysis [20]. Surface color is usually used as a maturity indicator of fruits; nevertheless, a slight variation in pH might cause considerable changes on the intensity of fruit color [27]. Moreover, fruits may exhibit the same color but have varying anthocyanin content, as the quantification of anthocyanin content is related to fruit size and water content, as well as anthocyanin distribution. 

Reports on the effect of cultivar on phytochemical content vary among different studies. For example, Mathieu et al. reported higher anthocyanin and phenolic content of ‘Nova’ than ‘York’ during a two-year observation [20], consistent with our findings, while an opposite observation was reported by Perkins-Veazie et al. [19]. Conflicting results may also occur within the same study, as Lee and Finn observed significantly higher anthocyanins content of ‘Adams’ than ‘Johns’ and ‘York’ in 2005, but not in 2004 [1]. Such variability suggests an interplay involving both genetics and environmental factors. Geographic, climatic, edaphic conditions, and even the position of the sampled fruits on the mother-plants can all be origins of the variance displayed [27]. American elderberry appeared to be highly responsive to these environmental factors; thus, cultivar selection with interested biochemicals should be in accordance with specific environmental conditions and cultivation approaches. 

American elderberry fruits went through significant phytochemical changes during ripening, which was reflected by increased °Brix, pH, phenolic (including anthocyanins) content, and decreased percentage of polymeric color. During red fruit ripening, an increase in °Brix and pH is the most common, along with an accumulation of anthocyanins. The increased °Brix and pH are attributed to the acids in the fruit converting to sugars during the ripening process [2,27]. Although both increased significantly, the total phenolic content did not increase as much as the anthocyanin content did. This phenomenon was also observed during the development of other berries, like grape and raspberry [28,29]. The total phenolic content is an equilibrium between biosynthesis and metabolism, thus can ascend, decline, or stay flat during maturation depending on enzyme activities and precursor availability [27]. On the other hand, anthocyanins are continuously synthesized during fruit development, thereby the accumulation of anthocyanin and phenolic is not expected to be correlated.

Acylated anthocyanins are a group of more stable anthocyanins and more commonly found in vegetable sources like red radish, black carrot, and red cabbage [13]. Conventional fruit sources, such as cranberry, blackberry, blueberry, or European elderberry usually only contain non-acylated anthocyanins [13]. Red grape may contain about 30% anthocyanins acylated with aromatic or aliphatic acids [30]. Black goji berry was reported to be abundant in anthocyanins acylated with *p*-coumaric acids [12], but it is mainly distributed in central and east Asia with limited availability. The high acylated anthocyanin content as well as its accessibility in North America make American elderberry an excellent candidate as a natural colorant with many attractive traits.

Both exogenous and endogenous factors can initiate anthocyanin composition evolution. For example, in blueberry, cyanidin derivatives were more abundant in ripe fruits, whereas malvidin derivatives were predominant in overripe fruits [27]; the stink bug infestation decreased malvidin-derivatives and increased other aglycone derivatives [31]. Our study revealed a higher ratio of acylated anthocyanins in American elderberry at the half-ripe stage (Figure 2). Similar fluctuation has been reported in some vegetables, such as colored waxy corn [32] or red cabbage [33], both featuring a higher acylated anthocyanin ratio at an earlier ripening stage. Fruit sources, like blueberry, usually express variation on anthocyanin aglycone.

Cyanidin-3-(*cis*)-coumaroylsambubioside-5-glucoside made up 7.6% and 1.8% of the total pigments in HRFE and FRFE, respectively (Figure 2). This isomer differed from its *trans* counterpart only on the spatial configuration of the acyl group but occurs much more rarely in nature with different UV-Vis spectral characteristics. An absorption band is generally observed in the 310–360 nm range for anthocyanins acylated with aromatic acids, giving a higher ratio of A_310–360_/A_vis-max_ than for aliphatic acylated or non-acylated anthocyanins [13]. However, our UV-Vis spectra of *cis* isomers revealed a much lower absorption in that range, being approximately half of A_310_/A_vis-max_ ratio of *trans* isomers. Apart from that, the *cis* isomers displayed a larger *l*_vis-max_ (525 nm) than their *trans* counterparts (521 nm) in the same solvent. The coexistence of *cis* and *trans* isomers are seldom found in edible sources, and current studies about the impact of *cis-trans* configuration on the color of these pigments are few and limited only to petunidin or delphinidin derivatives [34]. Therefore, our research provided novel information about the impact of acyl group spatial configuration on cyanidin derivatives.

Different anthocyanin/polyphenol ratios between the HRFE and FRFE can explain their spectrophotometric and colorimetric differences. The anthocyanin/phenolic ratio increased from ~20% to 85% from half- to fully-ripened. Therefore, when both extracts were standardized by their anthocyanin concentration, the HRFE contained significant higher polyphenol levels. The main phenolics in the extracts besides anthocyanins were hydroxycinnamic acids and flavonols (data not shown). These compounds can affect the extract color expression via producing yellow colors on their own (*l*_vis-max_ ~360 nm), or anthocyanin copigmentation. Though the copigmentation may enhance anthocyanin stability, such interaction is only efficient with non-acylated anthocyanins at a low acidic pH [35], and may alter the color properties simultaneously. With the removal of these interfering compounds, the American elderberry pigments expressed more vivid colors and obtained a sharper spectrum.

Due to the high similarities of their anthocyanin profiles, different cultivars produced similar color with most pairwise ΔEs < 5. All of the extracts were able to express blue hues at pH 8 with h* between 233.1–253.6°, resembling that of FD & C Blue No.2 [33]. About 90% of pigments in American elderberry were cyanidin-3,5-diglycosylated (Figure 1). This glycosidic pattern is characterized by a larger *l*_vis-max_ than cyanidin-3-glycosides at all pH, therefore is capable of expressing blue hues at alkaline pH [26]. Common fruit-based anthocyanin sources like European elderberry and chokeberry lack this glycosidic pattern and do not express blue hues at any pH. Currently, natural sources of blue colorants are very limited, thus the glycosidic pattern along with the high acylated anthocyanin content make American elderberry a desirable natural colorant candidate.

## 4. Materials and Methods

### 4.1. Reagents

ACS or HPLC grade acetone, chloroform, methanol, trifluoroacetic acid (TFA), ammonium hydroxide (NH_4_OH), acetonitrile, potassium hydroxide (KOH), and sodium phosphate dibasic (Na_2_HPO_4_) were purchased from Fisher Scientific (Fair Lawn, NJ, USA). ACS grade formic acid was purchased from Honeywell (Morris Plains, NJ, USA).

### 4.2. Collection of Plant Materials

American elderberry fruits of cultivars ‘Adams’, ‘Johns’, ‘Nova’, ‘Wyldewood’ and ‘York’ were harvested from plants grown at the South Center, The Ohio State University, near Piketon, Ohio, USA, during two summers in 2015 and 2017. Fruits harvested between mid-August and early-September 2015 were used to investigate the impact of cultivar. ‘Nova’ and ‘Wyldewood’ fruits were further harvested in late-August 2017 to determine the impact of ripeness.

Fruit samples were harvested from three plants of each cultivar. After the harvest, samples were placed in polyethylene bags, labeled, and transported to the lab. Fruits were stored at −20°C until further analysis.

### 4.3. Determination of Maturity Stage

**Figure 4:**
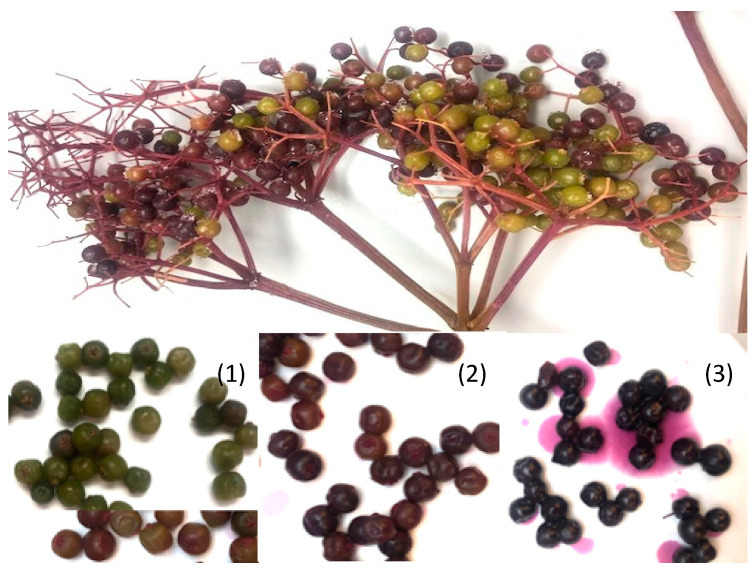
American elderberry fruits on the same branch at (**1**) Immature, (**2**) Half-ripe, and (**3**) Fully-ripe stages.

Elderberry fruits on a branch do not mature at the same time (Figure 4); thus, individual fruits were sorted into one of three maturity categories according to their appearance and surface color before analysis: (1) Immature stage: fruits were entirely green or with minimal red color shown on the surface; (2) Half-ripe stage: fruits were overall red with minimal green color shown on the surface; (3) Fully-ripe stage: fruits were entirely dark purple-violet on the surface (Figure 4). Surface color properties (Hunter CIE L*, a*, b*) of samples in categories (2) and (3) were measured by a Minolta handheld colorimeter (Konica Minolta, Osaka, Japan). The L* value indicated the level of lightness with 0 representing the darkest black and 100 representing the brightest white; the a* value indicated the redness (+) and greenness (−) of the object and b* value indicated the yellowish (+) and bluish (−) of the objective, according to the International Commission on Illumination (CIE) [36]. To determine the impact of cultivar, only berries in category (3) were retained to minimize the variance in maturity. To determine the impact of ripeness, berries in category (2) and (3) were stored separately for further analysis.

### 4.4. Fruit Extracts and Their Quality Attributes

°Brix and pH were measured during sample extraction. Each sample was weighed (~30 g) and blended with liquid nitrogen for 2 min. °Brix was quantified using a handheld refractometer (Atago Co., Ltd., Tokyo, Japan), and pH was measured using a pH meter (Mettler Toledo, Inc., Columbus, OH, US) after the powder was thawed into puree.

Anthocyanins and other phenolics were extracted with acidified acetone and partitioned with chloroform [37]. About 30 mL of acetone acidified with 0.01% HCl was added to the puree and homogenized for 2 min. The blend was then vacuum filtered using a Buchner funnel with Whatman #4 filter paper (Whatman Inc, NJ, US). After filtration, the slurry was re-extracted with 70%(*v*/*v*) aqueous acetone with 0.01% HCl until a pink color was barely visible. The anthocyanin extract was placed in a separatory funnel with 2 volumes of chloroform, and the mixture was gently mixed and left to sit at room temperature until a good separation was achieved. The top layer (anthocyanin and phenolic concentrate) was collected into a flask, while the bottom layer (chloroform and polar solvents) was discarded. Residual acetone was evaporated using a Buchi rotary evaporator at 40 °C. The final volume of sample was documented for quantification purposes.

### 4.5. Quantification of Anthocyanin and Phenolic Content

Monomeric anthocyanin content was estimated using the pH differential method [38]. The absorbance of the anthocyanin extracts at pH 1 and pH 4.5 was measured using a SpectraMax 190 Microplate Reader (Molecular Devices, Sunnyvale, CA, USA) at 520 nm (*λ*_max_) and 700 nm with automated 1-cm pathlength correction. The molecular weight (449.2 g mol **) and molar extinction coefficient (29,600 L cm ** mol **) of cyanidin-3-glucoside (C3G) were used for calculation. The total monomeric anthocyanin content was expressed as mg C3GE per 100 g of FW.

Polymeric color was determined by measuring the absorbance of the extracts at 420 nm, 520 nm (*λ*_max_), and 700 nm after being treated with sodium bisulfite [38]. The percent polymeric color was expressed as the ratio between polymerized color and overall color density. 

Total phenolic content was quantified using the Folin-Ciocalteau method and expressed as gallic acid equivalents [39]. Absorbance was read at 765 nm using the SpectraMax 190 Microplate Reader. Total phenolic content was calculated and expressed as mg gallic acid equivalents (GAE) per 100 g of FW.

### 4.6. Sample Purification

Anthocyanin extracts were semi-purified using solid phase extraction with a C-18 cartridge. The C-18 cartridge was activated by methanol before loading the elderberry crude extract. The crude extract was then washed with acidified water (0.01% *v*/*v* HCl) and ethyl acetate to eliminate acids, sugars, and less polar phenolics. The semi-purified anthocyanins were eluted with acidified methanol (0.01% *v*/*v* HCl) and evaporated until dryness to remove all methanol. Anthocyanins were re-dissolved in acidified water (0.01% *v*/*v* HCl) for further analysis.

### 4.7. Anthocyanin Identification

Anthocyanin identification was conducted using a Shimadzu ultra-High-Pressure Liquid Chromatography (uHPLC) system equipped with LC-2040C pumps coupled to a triple-quadrupole Shimadzu LCMS-8040 mass spectrometer using LC-2040 PDA detector (Shimadzu, Columbia, MD, USA). A Restek reverse phase C-18 column (50 × 2.1 mm) with 1.9 µm particle size was used (Restek Corporation, Bellefonte, PA, USA). Samples were filtered through a 0.2 µm RC membrane filter (Phenomenex, Torrance, CA, USA) before injection (10 µL). Samples were run using a flow rate of 0.25 mL/min and solvent A: 4.5% formic acid in HPLC water and solvent B: 100% acetonitrile, at 60 °C. Anthocyanin separation was achieved using a linear gradient from 1% to 3% B in 2 min; 2 to 3 min, 3% to 4.5% B; 3 to 7.5 min, 4.5% to 8.5% B; 7.5 to 13 min, 8.5% to 40% B. Spectra (200–700 nm) was collected. The mass spectrometer was set for positive ion mode, with total ion scan from 100–1000 m/z and precursor ion scan at 271, 287, 301, 303, 317, and 331 m/z. MS data, order of elution, and comparison to literature were used for the anthocyanin identification.

### 4.8. Buffer and Sample Preparation

Buffer solutions of pH 2–9 were prepared as follows: 0.025 M KCl for pH 2, 0.1 M sodium acetate for pH 3–6, 0.2 M Na_2_HPO_4_ and 0.2 M NaH_2_PO_4_ for pH 7–9 [12,40]. The final anthocyanin concentration was adjusted to 100 mM with buffer and kept at 4 °C in dark.

### 4.9. Spectrophotometric and Colorimetric Analysis

The initial spectral measurement (400–700 nm, 1 nm interval) of each extract at pH 2–9 was taken 1 h after mixing with buffers, when sufficient equilibration was achieved. A 300 mL aliquot was pipetted into a poly-D-lysine coated polystyrene 96-well microplate and read on the SpectraMax 190 Microplate Reader. The spectral data was converted into colorimetric data (L* (lightness), a*, b*, C*_ab_ (chroma), h*_ab_ (hue angle)) using the ColorBySpectra software according to CIE 1964 standard observer, D65 illuminant spectral distribution and 10° observer angle [41]. The color difference (ΔE) was calculated using the following equation:

√(Δa** + Δb** + ΔL**)

### 4.10. Statistical Analysis

One-way ANOVA (two-tailed, *a* = 0.05) and post hoc Tukey test were conducted to determine the impact of cultivar. A *t*-test was conducted to evaluate the impact of ripeness. All of the statistical analysis was conducted using Prism software (GraphPad, La Jolla, CA, USA).

## 5. Conclusions

American elderberry differed on most phytochemical attributes, including pH, anthocyanin content and profile, as well as anthocyanin/phenolic ratio. Those differences led to small but visible color and spectral differences under various pH environments, particularly under alkaline pH. Johns’ berries exhibited overall higher anthocyanin content and acylated anthocyanin proportion with relatively low non-anthocyanin phenolic content, possessing more favorable attributes for potential application as natural colorants. All these attributes increased during fruit ripening, except the percentage of polymeric color and acylated anthocyanin. The acylated anthocyanin proportion dropped from 80% at the half-ripe stage to 70% when fully ripened. All the major anthocyanins in American elderberry were cyanidin-derivatives, with both *cis* and *trans*-configured *p*-coumaric acid acylation co-existing. FRFE exhibited a “red-colorless-purple-blue” color expression pattern at pH 2–9 and expressed more vibrant colors and sharper spectra after C-18 semi-purification. 

Our results contribute to the selection of proper cultivars and ripeness for specific applications of this North American native plant and expand the potential scientific and industrial applications. The high proportion of acylated anthocyanins, along with blue hues (*λ*_vis-max_ ~600 nm) expression of its extracts under alkaline pH, make it a promising alternative to synthetic dyes and expands the natural color spectrum. This is particularly attractive for food applications as most fruit sources contain little to no acylated anthocyanins and tend to be less stable. On the other hand, vegetable sources with high levels of acylated anthocyanins typically possess stronger flavor. Moreover, *trans* and *cis*-configured cyanidin-derivatives co-exist in American elderberry, which is rare in nature. Thus, this material can be further utilized to explore the impact of acyl group spatial configuration on anthocyanin color properties.

## Figures and Tables

**Table 1:** Fruits’ phytochemical attributes comparison among different American elderberry cultivars. Results expressed as mean ± SD (*n* = 3). Cultivars with different superscripts were significantly different (*p* < 0.05).

Cultivar	°Brix	pH	Mono ACN ^1^	Poly Color ^2^	TP ^3^	ACN/TP ^4^
Adams	12.9 ± 1.1 ^a^	4.7 ± 0.11 ^a,b^	499 ± 91 ^a,b^	7.5 ± 0.8 ^a,b^	684 ± 88 ^a^	73 ± 4 ^a^
Johns	12.0 ± 1.1 ^a^	4.8 ± 0.05 ^b^	595 ± 26 ^b^	5.0 ± 0.5 ^a^	706 ± 22 ^a^	84 ± 4 ^b^
Nova	12.4 ± 0.3 ^a^	4.8 ± 0.06 ^b^	456 ± 73 ^a,b^	9.1 ± 1.5 ^a,b^	700 ± 77 ^a^	65 ± 4 ^a^
Wyldewood	12.0 ± 1.1 ^a^	4.5 ± 0.08 ^a^	471 ± 36 ^a,b^	7.3 ± 0.4 ^a,b^	637 ± 51 ^a^	74 ± 2 ^a,b^
York	13.1 ± 0.4 ^a^	4.9 ± 0.12 ^b^	354 ± 59 ^a^	10.8 ± 1.1 ^b^	582 ± 52 ^a^	61 ± 3 ^a^

^1^ Monomeric anthocyanin (mg cyanidin-3-glucosides equivalent/100 g fresh weight); ^2^ Polymeric color (%); ^3^ Total phenolics (mg gallic acid equivalent/100 g fresh weight); ^4^ Anthocyanin/Total phenolic (%).

**Table 2:** Percentage of individual anthocyanin in different American elderberry cultivars. Anthocyanins are listed in the order of elution. Results expressed as mean ± SD (*n* = 3). Cultivars with different superscripts were significantly different (*p* < 0.05).

Peak		Adam	Johns	Nova	Wyldewood	York	Overall
**Non-acylated**	1	7.2 ± 0.7 ^b^	7.9 ± 0.7 ^b^	8.2 ± 0.7 ^b^	8.5 ± 0.5 ^b^	5.2 ± 0.2 ^a^	7.4 ± 1.3
	2	16.2 ± 2.3 ^a^	15.4 ± 1.1 ^a^	16.6 ± 1.6 ^a^	18.6 ± 0.5 ^a^	16.1 ± 0.7 ^a^	16.6 ± 1.7
	Sum	23.3 ± 3 ^a^	23.3 ± 1.8 ^a^	24.8 ± 2.2 ^a^	27.2 ± 1.0 ^b^	21.3 ± 0.9 ^a^	24.0 ± 2.6
**Acylated**	3	2.3 ± 0.2 ^b^	1.7 ± 0.1 ^a^	1.8 ± 0.0 ^a^	1.9 ± 0.1 ^a^	2.3 ± 0.2 ^b^	2.0 ± 0.3
	4	2.4 ± 0.2 ^b^	2.1 ± 0.1 ^b^	2.4 ± 0.2 ^b^	1.5 ± 0.1 ^a^	2.1 ± 0.3 ^b^	2.1 ± 0.4
	5	67.1 ± 2.7 ^b^	67.0 ± 2.3 ^b^	64.1 ± 2.5 ^a,b^	59.8 ± 0.6 ^a^	67.0 ± 1.8 ^b^	65.0 ± 3.5
	Sum	71.9 ± 2.4 ^b^	70.8 ± 2.2 ^b^	68.3 ± 2.6 ^a,b^	63.2 ± 0.6 ^a^	71.4 ± 1.6 ^b^	69.1 ± 3.7

**Table 3:** Spectrophotometric (*l*_vis-max_, nm) and colorimetric (CIE-L*, C*, h*) data of half and fully-ripe ‘Nova’ extracts before and after C-18 semi-purification in pH 2–9 buffers (*n* = 3). Data was collected 1 h after mixing.

Extracts		pH2	pH3	pH4	pH5	pH6	pH7	pH8	pH9
**Lambda Max (** ***l*_max_, nm)**									
Half-ripe	CE ^1^	520 ± 1	515 ± 1	ND ^2^	ND	ND	ND	593 ± 2	580 ± 0
	C18	519 ± 3	522 ± 1	516 ± 3	ND	ND	540 ± 3	596 ± 1	577 ± 5
Fully-ripe	CE	519 ± 2	524 ± 3	523 ± 1	529 ± 9	528 ± 2	549 ± 1	600 ± 0	600 ± 0
	C18	520 ± 0	522 ± 0	524 ± 3	527 ± 1	523 ± 6	551 ± 2	597 ± 1	597 ± 1
L* (Lightness)									
Half-ripe	CE	54.8 ± 0.3	59.8 ± 0.1	64.2 ± 1.5	66.9 ± 0.1	68.0 ± 0.1	66.0 ± 0.3	54.6 ± 0.6	54.6 ± 0.4
	C18	55.5 ± 0.1	60.0 ± 0.5	70.5 ± 0.4	73.1 ± 0.3	74.1 ± 2.3	56.3 ± 0.4	35.5 ± 0.4	54.9 ± 0.5
Fully-ripe	CE	64.0 ± 0.7	75.6 ± 0.4	85.4 ± 0.3	88.1 ± 0.3	88.3 ± 0.2	83.0 ± 0.3	73.8 ± 0.3	73.4 ± 0.7
	C18	63.7 ± 0.1	73.0 ± 0.2	85.1 ± 0.1	87.3 ± 0.5	87.8 ± 0.7	58.3 ± 0.9	39.4 ± 0.1	45.3 ± 0.4
C* (Chroma)									
Half-ripe	CE	50.8 ± 0.5	40.5 ± 0.2	33.7 ± 0.3	32.5 ± 0.1	31.4 ± 0.3	30.3 ± 0.3	27.0 ± 0.5	48.9 ± 0.4
	C18	67.5 ± 0.1	52.6 ± 0.2	30.7 ± 0.1	26.3 ± 0.2	25.5 ± 2.1	22.4 ± 0.1	7.4 ± 0.4	46.5 ± 0.5
Fully-ripe	CE	52.6 ± 0.3	28.6 ± 0.3	8.1 ± 0.2	3.2 ± 0.2	2.5 ± 0.2	2.7 ± 0.3	17.5 ± 0.2	14.3 ± 0.6
	C18	66.0 ± 0.1	45.7 ± 0.5	15.4 ± 0.2	7.7 ± 0.2	7.1 ± 0.2	34.4 ± 0.7	41.5 ± 0.4	30.9 ± 0.2
h* (Hue Angle)									
Half-ripe	CE	31.9 ± 0	42.5 ± 0.1	58.0 ± 0.3	67.4 ± 0.2	70.8 ± 0.2	72.2 ± 0.2	103.1 ± 0.1	88.3 ± 0.4
	C18	23.9 ± 0.1	23.2 ± 0.3	44.6 ± 0.3	60.5 ± 0.3	64.7 ± 1.2	32.4 ± 0.4	168.0 ± 4.6	85.5 ± 0.5
Fully-ripe	CE	1.1 ± 0.6	356.9 ± 0.2	7.5 ± 0.7	36.2 ± 1.4	51.6 ± 0.8	341.8 ± 4.0	249.2 ± 0.3	153.4 ± 1.0
	C18	9.9 ± 0.1	1.4 ± 0.1	10.9 ± 0.6	28.9 ± 0.6	36.7 ± 1.6	316.2 ± 0.5	270.4 ± 0.4	238.1 ± 0.2

^1^ Crude extract. ^2^ Not detected.

**Table 4:** Mean (*n* = 3) color differences (∆E) of the extracts from different cultivars of fully-ripe fruits at pH 2–9.

	Johns	Nova	Wyldewood	York
**Adam**				
pH2	1.25	4.61	0.91	3.31
pH3	0.81	4.34	1.60	8.05
pH4	1.09	0.86	0.17	3.52
pH7	1.18	2.18	1.61	4.38
pH8	6.06	6.11	8.78	14.44
**Johns**				
pH2		5.34	1.81	4.21
pH3		4.79	2.16	8.21
pH4		1.75	1.05	3.60
pH7		2.71	12.13	4.62
pH8		1.49	3.00	9.21
**Nova**				
pH2			3.96	3.69
pH3			2.92	8.25
pH4			0.78	3.30
pH7			2.48	2.40
pH8			2.75	8.59
**Wyldewood**				
pH2				2.50
pH3				7.28
pH4				3.41
pH7				3.97
pH8				6.73

## Abbreviations

ACN	Anthocyanin
Cou	Coumaroyl
Cy	Cyanidin
FRFE	Extract from fully-ripe fruits
Glu	Glucoside
HPLC	High-performance liquid chromatography
HRFE	Extract from half-ripe fruits
Mono	Monomeric
MS	Mass spectroscopy
ND	Not detected
Poly	Polymeric
Sam	Sambubioside
TP	Total phenolics
